# Risk factor clustering in men and women with CHD in the Southern Cone of Latin America

**DOI:** 10.1016/j.ijcrp.2023.200172

**Published:** 2023-01-13

**Authors:** Anna Marzà-Florensa, Laura Gutierrez, Pablo Gulayin, Ilonca Vaartjes, Diederick E. Grobbee, Kerstin Klipstein-Grobusch, Vilma Irazola

**Affiliations:** a Julius Global Health, Julius Center for Health Sciences and Primary Care, University Medical Center Utrecht, Utrecht University, Utrecht, the Netherlands; b Instituto de Efectividad Clínica y Sanitaria, Buenos Aires, Argentina; c Division of Epidemiology and Biostatistics, School of Public Health, Faculty of Health Sciences, University of the Witwatersrand, Johannesburg, South Africa

**Keywords:** Secondary prevention, Sex differences, Cardiovascular RF, Education

## Abstract

**Background:**

Presence of multiple risk factors (RF) increases the risk for cardiovascular morbidity and mortality, and this is especially important in patients with coronary heart disease (CHD). The current study investigates sex differences in the presence of multiple cardiovascular RF in subjects with established CHD in the southern Cone of Latin America.

**Methods:**

We analyzed cross-sectional data from the 634 participants aged 35–74 with CHD from the community-based CESCAS Study. We calculated the prevalence for counts of cardiometabolic (hypertension, dyslipidemia, obesity, diabetes) and lifestyle (current smoking, unhealthy diet, low physical activity, excessive alcohol consumption) RF. Differences in RF number between men and women were tested with age-adjusted Poisson regression. We identified the most common RF combinations among participants with ≥4 RF. We performed a subgroup analysis by educational level.

**Results:**

The prevalence of cardiometabolic RF ranged from 76.3% (hypertension) to 26.8% (diabetes), and the prevalence of lifestyle RF from 81.9% (unhealthy diet) to 4.3% (excessive alcohol consumption). Obesity, central obesity, diabetes and low physical activity were more common in women, while excessive alcohol consumption and unhealthy diet were more common in men. Close to 85% of women and 81.5% of men presented with ≥4 RF. Women presented with a higher number of overall (relative risk (RR) 1.05, 95% CI 1.02–1.08) and cardiometabolic RF (1.17, 1.09–1.25). These sex differences were found in participants with primary education (RR women overall RF 1.08, 1.00–1.15, cardiometabolic RF 1.23, 1.09–1.39), but were diluted in those with higher educational attainment. The most common RF combination was hypertension/dyslipidemia/obesity/unhealthy diet.

**Conclusion:**

Overall, women showed a higher burden of multiple cardiovascular RF. Sex differences persisted in participants with low educational attainment, and women with low educational level had the highest RF burden.

## Highlights

• Women with CHD had a higher burden of multiple RF compared to men.
• Sex differences were marked in low educated subjects.

## 1 Introduction

There are relevant differences in coronary heart disease (CHD) burden, mortality, and treatment between men and women [1]. Sex differences exist also in cardiovascular risk factors (RF): some RF such as smoking and hypertension are reported to be more common in men, while obesity and diabetes are more prevalent in women [[2], [3], [4]]. Cardiovascular RF tend to cluster in individuals, and the presence of multiple RF increases the risk of cardiovascular disease more than the added risks of individual RF [5]. Studies from different world regions, studying the presence from 4 to 12 cardiovascular RF, show that the prevalence of multiple cardiovascular RF is high and ranges from 45.2% to 99.9% in men and 24.6%–99.8% in women [[4], [5], [6], [7], [8]], although these estimates depend on how many and which RF are included in the analysis. The evidence on sex differences in RF clustering shows diverging results, with studies finding a higher burden of multiple RF in women [7,9] and others in men [4,5,8]. Generally, these clusters are composed of various RF combined with obesity and diabetes in women, and smoking in men [7,10]. Socioeconomic status is also known to influence the presentation of multiple cardiovascular RF, with people with lower educational attainment being more prone to present multiple cardiovascular RF [11]. Besides, the magnitude and direction of sex differences in the burden of multiple cardiovascular RF can vary by socioeconomic status [11,12]. Information about RF clustering in coronary heart disease (CHD) patients is scarce but it shows that prevalence of multiple RF is very high in CHD patients, especially in men [2]. Since this population is at very high cardiovascular risk [13], a better understanding of clustering of CVD RF in men and women with CHD will contribute to the development of effective secondary prevention strategies. Within the South American region, available studies reporting on RF clustering are limited [6,14,15]. The Centro de Excelencia en Salud Cardiovascular para el Cono Sur (CESCAS) study aims to research RF and CVD in 4 cities in the Southern Cone of Latin America [3]. More than two thirds of the CESCAS overall study population presented with ≥3 cardiovascular RF, and more women than men had ≥5 [14]. Nevertheless, information about RF clustering in South American men and women with established CHD is currently not available. The current study aims to investigate differences in the distribution of multiple RF between men and women with established CHD in the southern Cone of Latin America, within CESCAS Study. In addition, we plan to describe potential sex differences in RF clustering across educational levels.

## 2 Methods

The CESCAS study is a population-based cohort including 7524 participants aged 35–74 years. The details of the study design and sampling methods have been described previously [3]. Shortly, urban and rural participants were recruited from randomly selected samples between February 2010 and December 2011 in 4 medium-size cities in the Southern Cone of Latin America: Bariloche and Marcos Paz (Argentina), Temuco (Chile) and Pando-Barros Blancos (Uruguay). The baseline cross-sectional data obtained in the first phase of CESCAS was used for this analysis. For this study, we focus on the 634 participants with a diagnosis of CHD.

The study was conducted following the guidelines for protection of human volunteers’ rights and it is in compliance with the Declaration of Helsinki. The protocol of the study received approval from Institutional Review Boards for all participating institutions in Argentina, Chile, Uruguay and the United States [3].

Data collection took place in a home visit and through clinical examination. During the home visit, a standard questionnaire was used to collect data on sociodemographic characteristics (including age, sex and educational level), history of cardiovascular disease and RF (including CHD, hypertension, diabetes, dyslipidemia, and treatment for these conditions), lifestyle RF (such as cigarette smoking, alcohol consumption, physical activity and diet).

Physical activity was assessed using the International physical activity questionnaire-short form [16]. The activities registered in the questionnaire were converted into metabolic equivalents (METs). The food frequency questionnaire used to collect nutritional information was adapted from the NCI Dietary History Questionnaire and validated in the Argentina, Chile and Uruguay [17,18].

Blood pressure and anthropometric variables were measured during the clinical examination using standardized procedures. Blood pressure was measured 3 times with standard mercury or aneroid sphygmomanometers. Participants were on a sitting position after 5 min of rest for the blood pressure measurements, and the mean of the 3 readings was used for analysis. Body weight, height and waist circumference were measured twice and the mean of the two values was used for the analysis. Body weight was measured with standing scales and height was measured with stadiometers. Waist circumference was measured at 1 cm above the navel at minimal respiration.

Lipids and glucose were measured from overnight fasting blood samples. Blood glucose, total cholesterol, HDL-cholesterol and triglycerides were measured with standard methods, and LDL-cholesterol was calculated with the Friedewald equation if participants had a triglyceride level <400 mg/dL [19].

CHD was defined as self-reported previous acute myocardial infarction (MI), angina or coronary procedure, determined by the following questions during an interview with trained staff: “Has a doctor ever said that you have angina?”, “Has a doctor ever said that you had a heart attack?” and “Have you had a balloon angioplasty, a stent, or bypass surgery to the arteries in your heart to improve the blood flow to your heart?”.

Hypertension was defined as mean systolic blood pressure (BP) ≥140 mmHg and/or diastolic BP ≥ 90 mm Hg and/or current use of antihypertensive medication. Dyslipidemia was determined as total cholesterol ≥240 mg/dL,LDL-cholesterol ≥160 mg/dL, HDL-cholesterol <40 mg/dL, triglyceride ≥200 mg/dL or use of lipid-lowering medication. Participants were considered diabetic if they reported to have diabetes, if they presented fasting glucose levels ≥126 mg/dL, or if they used hypoglycemic medication. Obesity was defined as BMI ≥30 kg/m^2^ [14], and central obesity was determined at waist circumference ≥102 cm (women) and ≥88 cm (men) [14,20].

Alcohol consumption was considered excessive for men reporting >14 units per week or >5 units at one occasion (around 2 h more than once per month), and women reporting >7 units per week or >4 units in one occasion [21]. Alcohol units were considered as drink-equivalents containing 14 g of alcohol [22]. Low physical activity was defined as <600 MET-minutes/week of total physical activity [23], and low consumption of fruit and vegetables was defined as <5 servings per day (<400 g per day) [14].

Educational level was categorized by the highest level attained: primary, secondary or university [24].

We assessed RF clustering as counts and combinations of RF. RF count was determined as the number of cardiovascular RF each participant presented, ranging from zero to eight. Combinations of factors were treated as dichotomous variables and were defined as the simultaneous presence of each combination of 3 RF (among subjects with 3 RF), and of each combination of 4 RF (among subjects with ≥4 RF). We included 4 cardiometabolic (hypertension, dyslipidemia, diabetes and obesity or central obesity) and 4 lifestyle (current cigarette smoking, excessive alcohol consumption, low physical activity and unhealthy diet) RF for this analysis.

Categorical variables were presented as number of participants and percentage, and numerical variables as mean and standard error (SE). Age-adjusted RF prevalence estimates were calculated with the overall 2010 population distribution in the Southern Cone of Latin America as reference population [14].

Differences in the count of RF between men and women were tested in univariable analysis using *t*-test, and in a multivariable analysis adjusted by age with a Poisson regression model. Statistical significance was considered if p < 0.05. Furthermore, we performed subgroup analysis to explore sex differences in RF counts and combinations within educational levels.

All analyses were conducted with the statistical software RStudio [25] and the R package “survey” [26].

## 3 Results

There were 7524 respondents from the 10,554 randomly invited participants. Across the 4 study locations the response rate (73.4%) was similar in men and women. The prevalence of established CHD was 8.4% in the overall study population, 10.0% in men and 7.3% in women (634 participants with CHD).

Half of the participants with CHD were women, and mean age was 60.0 years (standard error 9.8 years). More than half of the participants completed primary school, 33.6% achieved secondary schooling, and 12.3% had a tertiary level or university degree. The most common presentation of CHD was angina, followed by myocardial infarction and history of coronary procedures, which were significantly more common in men (Table 1).

**Table 1:** Characteristics of the study population

Variable	Male	Female	Overall	p
**N**	317	317	634	
**Age (mean (SE))**	60.4 (0.39)	59.6 (0.56)	60.4 (0.53)	0.301
**Age group (%)**				
35–44	26 (8.2)	35 (11.0)	61 (9.6)	0.304
45–54	58 (18.3)	64 (20.2)	122 (19.2)	
55–54	115 (36.3)	95 (30.0)	210 (33.1)	
65–74	118 (37.2)	123 (38.8)	241 (38.0)	
**City (%)**				
Marzos Paz (Argentina)	76 (24.0)	55 (17.4)	131 (20.7)	0.089
Bariloche (Argentina)	64 (20.2)	63 (19.9)	127 (20.0)	
Temuco (Chile)	97 (30.6)	95 (30.0)	192 (30.3)	
Pando-Barros Blancos (Uruguay)	80 (25.2)	104 (32.8)	184 (29.0)	
**Education**^a^**(%)**				
Primary school	164 (51.7)	179 (56.5)	343 (54.1)	0.281
Secondary school	108 (34.1)	105 (33.1)	213 (33.6)	
University	45 (14.2)	33 (10.4)	78 (12.3)	
**Unemployment (%)**	9 (2.8)	16 (5.0)	25 (3.9)	0.154
**CHD Diagnosis (%)**				
Myocardial infarction	112 (35.4)	88 (28.2)	200 (31.8)	0.052
Angina	243 (76.9)	252 (79.5)	495 (78.2)	0.429
Coronary procedure	97 (30.6)	49 (15.5)	146 (23.0)	<0.001
**Hypertension**^b^**(%)**	249 (78.5)	235 (74.1)	484 (76.3)	0.191
**Dyslipidemia**^c^**(%)**	226 (72.9)	212 (68.8)	438 (70.9)	0.266
**Diabetes**^d^**(%)**	76 (24.5)	98 (31.8)	174 (28.2)	0.044
**Obesity**^e^**(%)**	129 (41.0)	171 (54.3)	300 (47.3)	<0.001
**Central obesity**^f^**(%)**	149 (47.3)	249 (78.5)	398 (63.0)	<0.001
**Smoking (%)**				
Current	78 (24.6)	65 (20.5)	143 (22.6)	<0.001
Former	158 (49.8)	73 (23.0)	231 (36.4)	
**Excessive alcohol consumption**^g^**(%)**	22 (7.0)	5 (1.6)	27 (4.3)	0.001
**Low physical activity**^h^**(%)**	101 (31.9)	142 (44.8)	243 (38.3)	0.001
**Low fruit and vegetable intake**^i^**(%)**	278 (87.7)	240 (76.2)	518 (82.0)	<0.001

a Highest educational level attained by participants.

b Systolic blood pressure ≥140 mmHg, diastolic blood pressure ≥90 mmHg, or current use of antihypertensive medication.

c Total cholesterol ≥240 mg/dL, LDL-cholesterol ≥160 mg/Dl, HDL-cholesterol <40 mg/dL, triglyceride ≥200 mg/dL or current use of lipid-lowering medication.

d Self-reported diabetes, fasting glucose ≥126 mg/dL, or current use of hypoglycemic medication.

e Body mass index ≥30kg/m2.

f Waist circumference ≥102 cm (women); ≥88 cm (men).

g > 14 units/week or >5 units at one occasion (men); >7 units/week or >4 units in one occasion (women). Alcohol units: drink-equivalents containing 14 g of alcohol. One occasion: around 2 h more than once per month.

h < 600 MET-minutes/week of total physical activity.

i < 5 servings/day (400 g/day).

**Fig. 1:**
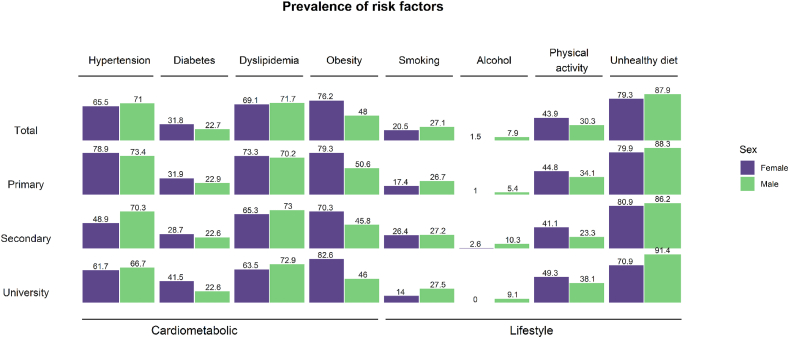
Prevalence of RF by type of RF and sex. **Footnote:** Results are expressed in percentages. Hypertension: systolic blood pressure ≥140 mm Hg, or diastolic blood pressure ≥90 mm Hg, or current use of antihypertensive medication Dyslipidemia: total cholesterol ≥240 mg/dL or/and LDL-cholesterol ≥160 mg/Dl, HDL-cholesterol <40 mg/dL, triglyceride ≥200 mg/dL or current use of lipid-lowering medication. Diabetes: self-reported diabetes, fasting glucose ≥126 mg/dL, or current use of hypoglicemic medication. Obesity: body mass index ≥30 kg/m^2^, or central obesity (waist circumference ≥102 cm in women ≥88 cm in men); alcohol units: drink-equivalents containing 14 g of alcohol; one occasion: around 2 h more than once per month). Low physical activity: <600 MET-minutes/week of total physical activity. Low fruit and vegetable consumption: <5 servings/day (400 g/day).

The prevalence of cardiometabolic RF ranged from 76.3% (hypertension) to 26.8% (diabetes), and the prevalence of lifestyle RF varied from 81.9% (unhealthy diet) to 4.3% (excessive alcohol consumption). Cardiometabolic RF as obesity, central obesity and low physical activity were more common in women, while the behavioral RF as smoking, alcohol consumption, and unhealthy diets were more prevalent in men (Table 1). RF prevalence by sex and educational level is presented in Fig. 1A.

**Fig. 2:**
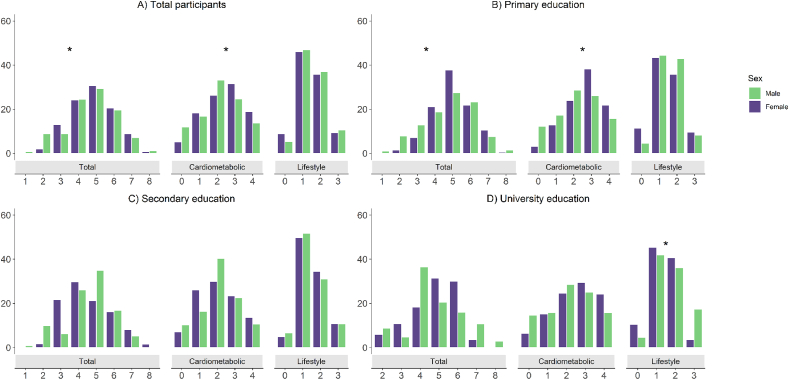
Number of overall, cardiometabolic and total RF in men and women by previous CHD history and educational level. **Footnote:** Cardiometabolic RF include hypertension, diabetes, dyslipidemia and obesity. Lifestyle RF include current smoking, excessive alcohol consumption, low physical activity, and unhealthy diet. *Indicates significant differences between men and women controlled by age.

Women presented with a higher number of RF than men (OR 1.05, 95% CI 1.02–1.08, Fig. 2A and Table 2). 84.9% of women and 81.5% of men presented ≥4 RF, and 60.7% of women and 57.1% of men presented ≥5 RF. By RF type, the burden of cardiometabolic RF was higher in women (OR 1.17, 95% CI 1.09–1.25) (Fig. 2A, Table 2) while that of lifestyle RF was similar in men and women.

**Table 2:** Results of Poisson-regression multivariable analysis. Results are expressed in rate ratios (95% confidence intervals).

	Overall	Primary education	Secondary education	University
**Outcome: number of RF**				
Female	1.05 (1.02–1.08)^a^	1.08 (1.03–1.12)^a^	1.04 (0.99–1.09)	1.00 (0.92–1.09)
Age group				
45-54	1.14 (1.07–1.21)^a^	1.06 (0.97–1.17)	1.28 (1.18–1.40)^a^	0.90 (0.76–1.06)
55-64	1.24 (1.17–1.31)^a^	1.16 (1.07–1.26)^a^	1.34 (1.24–1.47)^a^	1.14 (1.00–1.30)
65-74	1.25 (1.18–1.32)^a^	1.12 (1.11–1.29)^a^	1.31 (1.20–1.44)^a^	1.14 (1.00–1.29)
**Outcome: number of cardiometabolic RF**				
Female	1.17 (1.09–1.25)^a^	1.23 (1.13–1.34)^a^	1.09 (0.98–1.21)	1.18 (0.94–1.48)
Age group				
45-54	1.45 (1.22–1.74)^a^	1.19 (0.95–1.51)	1.92 (1.52–2.44)^a^	1.03 (0.54–1.98)
55-64	1.85 (1.57–2.20)^a^	1.59 (1.30–1.95)^a^	2.20 (1.75–2.79)^a^	1.75 (0.99–3.27)
65-74	1.88 (1.59–2.22)^a^	1.60 (1.32–1.96)^a^	2.29 (1.82–2.90)^a^	1.57 (0.88–2.94)
**Outcome: number of lifestyle RF**				
Female	0.95 (0.89–1.1.01)	0.92 (0.85–1.00)	1.03 (0.93–1.14)	0.80 (0.68–0.93)^a^
Age group				
45-54	0.97 (0.87–1.08)	0.99 (0.83–1.18)	1.03 (0.87–1.21)	0.72 (0.56–0.92)^a^
55-64	0.87 (0.78–0.96)^a^	0.82 (0.71–0.96)^a^	0.95 (0.81–1.12)	0.77 (0.62–0.96)^a^
65-74	0.89 (0.80–0.98)^a^	0.90 (0.78–1.05)	0.87 (0.73–1.04)	0.82 (0.66–1.02)

**Footnote:** Results are expressed in rate ratios (95% confidence intervals) by sex and controlled by age, in subjects with CHD in general and by educational level. Estimates represent the expected increase in count of RF in women compared to men controlled by age, in subjects with CHD in general and by educational level. Number of RF was treated as numeric outcome. Reference category for sex was male, and reference category for age group was 35–44 years.

a Indicates significant differences between men and women. Abbreviations: CHD (Coronary Heart Disease).

The most common RF combinations among subjects with ≥4 RF were hypertension/dyslipidemia/obesity/unhealthy diet (28.3% of men and 29.6% of women); and hypertension/diabetes/obesity/unhealthy diet (12.5% of men and 14.1% of women) (Table 3). Some combinations were more common in women: dyslipidemia/diabetes/low physical activity (9.8% and 7.1%, p = 0.04), and hypertension/diabetes/obesity/low physical activity (8.1% and 5.0%, p = 0.01 respectively). Combinations including smoking and excessive alcohol use were more frequent in men: hypertension/dyslipidemia/unhealthy diet/smoking (10.6% and 7.5%, p = 0.00), and hypertension/diabetes/obesity/excessive alcohol consumption (8.5% and 5.8%, p = 0.00) (Table 3). Supplementary Table 2 shows the prevalence of all combinations in men and women with at least 4 RF.

**Table 3:** Most common combinations of 3 and 4 RF in men and women by previous history of CHD and educational level. Results are expressed in percentages.

Participants with CHD (N = 634)				CHD and Primary education (N = 343)				CHD and Secondary education (N = 213)				CHD and University education (N = 78)			
Comb.	Male (N = 317)	Female (N = 317)	p	Comb.	Male (N = 164)	Female (N = 179)	p	Comb.	Male (N = 108)	Female (N = 105)	p	Comb.	Male (N = 45)	Female (N = 35)	p
HLOF	28.3	29.6	0.53	HLOF	31.4	40.0	0.17	HLOF	22.8	21.0	0.76	HLOF	33.2	25.4	0.55
HDOF	12.5	14.1	0.30	HDOF	14.7	18.5	0.42	HDOF	10.2	12.3	0.65	HLOP	22.6	13.5	0.34
HLOP	11.3	13.8	0.08	HLOP	12.9	18.3	0.21	HLOP	5.1	14.8	0.02	HDOF	11.9	15.7	0.72
LDP	7.1	9.8	0.04	LDP	8.3	12.9	0.25	LDP	5.4	11.7	0.10	LDP	8.7	14.8	0.45
HDOP	5.0	8.1	0.01	HDOP	5.5	12.3	0.04	HLFS	10.7	4.6	0.12	HDOP	8.5	9.1	0.92
HLFS	10.6	7.5	0.00	HLFS	11.2	5.1	0.04	HDOP	3.2	10.5	0.03	HLOS	6.6	5.2	0.78
LOFS	6.5	6.1	0.70	LOFS	8.8	7.1	0.61	AFL	7.8	2.6	0.13	HDOA	7.8	2.3	0.23
HDOA	8.5	5.8	0.00	HOFS	8.3	5.2	0.29	HDOA	6.3	3.4	0.31	AFH	9.1	0.0	0.09
HLOS	5.7	5.3	0.70	HDOA	10.7	2.7	0.00	LOFS	4.3	5.5	0.72	HLFS	8.6	0.0	0.12
HOFS	5.9	5.0	0.33	HLOS	7.7	5.1	0.37	HLOS	3.1	4.7	0.51	AFL	5.5	0.0	0.20
DOAS	0.6	0.3	0.17	HDPS	0.0	0.5	0.35								

**Footnote:** Results are expressed in percentages. Abbreviations: coronary heart disease (CHD), hypertension (H), dyslipidemia (L), diabetes (D), obesity (O), excessive alcohol consumption (A), smoking (S), low physical activity (P), low fruit and vegetable consumption (F). Education: highest educational level attained by participants. Hypertension: systolic blood pressure ≥140 mm Hg, diastolic blood pressure ≥90 mm Hg, or current use of antihypertensive medication Dyslipidemia: total cholesterol ≥240 mg/dL, LDL-cholesterol ≥160 mg/Dl, HDL-cholesterol <40 mg/dL, triglyceride ≥200 mg/dL or current use of lipid-lowering medication. Diabetes: self-reported diabetes, fasting glucose ≥126 mg/dL, or current use of hypoglycemic medication. Obesity: body mass index ≥30kg/m2, or central obesity (waist circumference ≥102 cm in women ≥88 cm in men). Excessive alcohol consumption: >14 units/week or >5 units at one occasion (around 2 h more than once per month) (men); >7 units/week or >4 units in one occasion (women). Alcohol units: drink-equivalents containing 14 g of alcohol. Low physical activity: <600 MET-minutes/week of total physical activity. Low fruit and vegetable consumption: <5 servings/day (400 g/day).

Women with primary education presented with a higher number of overall (RR 1.08, 95% CI 1.03–1.12) and cardiometabolic RF (RR 1.23, 95% CI 1.13–1.34) compared to men with the same educational level (Fig. 2B, Table 2). There were no significant differences in the overall number of RF by sex in participants with secondary education. Results from the subjects with tertiary education showed a lower number of lifestyle RF in women (RR 0.80 0.68–0.93) although this difference was not reflected in significant differences in the total RF number (Fig. 2D, Table 2). Those with a high educational attainment presented with a lower burden of RF compared to those with lower educational level. The percentages of men and women with 0–8 RF, respectively 0 to 4 cardiometabolic or lifestyle RF are detailed in Supplementary Table 1.

The most common RF combination in men and women with at least 4 RF in all educational groups was hypertension/dyslipidemia/obesity/unhealthy diet; followed by hypertension/diabetes/obesity/unhealthy diet in participants with primary and secondary schooling, and by hypertension/dyslipidemia/insufficient physical activity in participants with university education (Table 3).

Participants without a previous history of CHD had a lower RF burden and there were no significant sex differences in the number of RF. Results for CESCAS participants without a history of CHD are presented in Supplementary File 1.

## 4 Discussion

In this population-based study including 634 participants with CHD, we observed a high prevalence of cardiovascular RF individually and in clusters. Overall, women present with a higher number of cardiovascular RF. Sex differences in the number of RF were driven mainly by cardiometabolic RF, which were more common in women and more prevalent than lifestyle RF. There were marked sex differences in the number of RF among participants with low educational attainment, but these differences diluted for those with higher educational attainment. The most common combination of RF was hypertension/dyslipidemia/obesity/unhealthy diet.

Our study population was constituted by the same number of men and women. Studies on CHD patients often have a higher proportion of men due to the occurrence of the disease [27,28]. The equal proportion of men and women in our study may be explained by the higher occurrence of CHD in men, in combination with the higher participation of women in the study: 57.9% of participants from the overall CESCAS study population were female, and the prevalence of CHD was 6.8% in women and 7.4% in men.

We observed that some cardiovascular RF have a very high prevalence: more than three out of participants were hypertensive, four out of five had an unhealthy diet, more than half of the women were obese, and almost 80% of women had central obesity. The prevalence of most RF was comparable to those observed for Latin American men and women with CHD included in various studies. Higher prevalence estimates for hypertension (75.3%) were reported in the REACH Study, conducted in several Latin American countries [29]. The prevalence of obesity in the CESCAS study was higher than in REACH study (22.1%) and the Chilean registry GEMI (33.1%), but similar to the FNR study (46.0%, conducted in Uruguay) [[29], [30], [31]]. We observed high levels of central obesity, especially in women: in comparison a lower prevalence was observed in European centres in EUROASPIRE V (50.0%), while SURF CHD, with patients from 3 world regions, reported higher estimates in men (53.0%) and lower in women (68.0%) compared to the CESCAS study [27,32].

Clustering of RF was also very common: more than 80% of women and men had ≥4 cardiovascular RF. Overall, women had a higher burden of multiple RF. This finding is in line with previous literature [7,28,33,34], though some studies find a higher number of RF in men [35] and others don't find sex differences [5,8].

In our analysis, we show that the higher burden of RF in women is driven mostly by cardiometabolic RF. The higher burden of RF in women may be explained in part because RF that are usually more common in men, such as smoking and excessive consumption of alcohol, were relatively uncommon in our study population. Poortinga et al. [5] finds that men in the general adult population in England have a higher risk factor burden, and the study focuses on lifestyle factors only.

The higher burden of cardiovascular RF in women, especially cardiometabolic RF, may have several causes. Literature has reported that women have lower awareness of CHD and the importance of treatment. They also show lower adherence to cardioprotective medication [27,36] and to cardiac rehabilitation [11].

Our findings show that the most common combination of RF in participants with ≥4 RF is hypertension/dyslipidemia/obesity/unhealthy diet; followed by combinations that include hypertension and obesity combined with diabetes, dyslipidemia, unhealthy diet and low physical activity. Previous research found the most prevalent combinations in CHD patients in Iran were dyslipidemia/low physical activity and dyslipidemia/central obesity [7], while in healthy individuals in China the most common cluster in was hypertension/dyslipidemia/obesity [8]. This overlaps partly with our results, as dyslipidemia and obesity were components of the most common clusters. There were some differences, as hypertension was a component of many of the highly prevalent combinations in our study, but it was not part of the most frequent combinations in Iranian CHD patients. However, they calculated the prevalence of combinations of two RF, while we analyzed combinations of 3 and 4 RF.

We also observed sex differences in some of the most prevalent combinations. Wang et al. [10] compared RF clustering in Chinese and Dutch men and women, finding that the most frequent clusters included drinking in men and obesity in women among Chinese, and obesity and hypertension in Dutch women. In our study we found significant sex differences in some combinations including physical inactivity (more common in women), and alcohol use and smoking (more common in men). In both studies, excessive alcohol use was part of the most the frequent clusters in men. There were also some differences: smoking was part of the combinations that were more common in men in our study, but it was present in the highly prevalent clusters in both men and women in the Dutch and the Chinese populations. This is an example of how the composition of RF clusters, as well as the different cluster composition by sex, can have regional variations [10].

It is important to investigate the specific components of RF combinations, as it has been shown that different combinations have differential risks for cardiovascular mortality and morbidity [6,35,37]. Studies carried out in The Netherlands in individuals with high CVD risk and in a healthy population in Asia showed that combinations including hypertension [35,37] and smoking [35] are associated with higher risks for CVD events. This is of concern since in our study since hypertension was a component in most highly prevalent combinations in individuals with at least 4 RF. However, there are regional differences in the risks associated with individual combinations, and therefore it would be important to research the risk of cardiac events associated with individual RF combinations specifically for the Southern Cone of Latin America. Unfortunately, this is not possible in our study due to the cross-sectional nature of the data.

Cardiometabolic RF were more common than lifestyle RF in men and women in the CESCAS Study population. There were sex differences in the number of cardiometabolic RF, with women presenting a higher number, but not on the lifestyle RF. Previous literature also finds a predominance of cardiometabolic RF, although in this case they were more common in men [38].

The modification or control of cardiometabolic RF is dependent on lifestyle changes and medication use. In our results, although lifestyle factors had a lower burden than cardiometabolic RF, there was a high prevalence of unhealthy diet and low physical activity. Although there may be other factors at play, the fact that there are sex differences in cardiometabolic RF but not in lifestyle RF may indicate that the same unhealthy behaviors may result in a higher risk of chronic conditions in women. Several studies have found that behavioral RF impact women more strongly, and women have higher CHD morbidity and mortality from the same RF [36,39,40].

Other factors that could possibly contribute to the higher RF burden in women may relate to cardiovascular disease awareness, healthcare utilization, and medication use. Previous literature suggests that women show lower awareness of cardiovascular disease [27,41] and higher rates of healthcare services utilization in several world regions [42,43]. Use of cardioprotective medication is an important measure to control cardiovascular RF in secondary prevention of CHD. It is widely reported that women with CHD have lower medication rates [27,[44], [45], [46]] and less attendance to cardiac rehabilitation compared to men [47]. In the CESCAS Study, and more generally in Latin America, women presented with higher awareness and treatment levels for hypertension and diabetes [[48], [49], [50]]. However, these results refer to the general population, and are not specific for subjects with established CHD. Literature also points at a later age of diagnosis of CHD in women [11,27,36] as a potential explanation for the higher RF profile. The investigation of sex differences in RF awareness and treatment in CESCAS participants with CHD is beyond the scope of this analysis, but might provide further understanding of our results, and should be addressed in further studies.

Our analysis shows that sex differences were marked among participants with primary educational attainment and that women with lower educational attainment had the highest burden of RF. Literature shows that lower educational level is associated with presenting with a higher number of RF [5,8,33,34]. Previous studies have described that the association of educational level or other socioeconomic factors with a higher number of RF is stronger in women than in men [11,28,36,38,51]. In Argentina, Rodriguez et al. [12] describes an increase in the prevalence of most cardiovascular RF from 2005 to 2013, and that this increase was more pronounced among women with low educational levels. Our results add to previous studies showing that the double inequality of sex and educational level is also observed in subjects with CHD with high cardiovascular risk.

Our results have implications for research, health policies and clinical practice. In terms of research, it is important to study sex not as isolated determinant of cardiovascular health, but encompassed in the subject's socioeconomic circumstances. The findings of our study also highlight the need to tailor prevention and RF management strategies to specific population groups. Understanding potential drivers of RF burden and clusters (such as awareness, medication use and adherence), and how these determinants may vary according to different demographics of individuals with CHD, is important to design interventions that are effective in improving RF management, including patient education in lifestyle and medication use, screening, treatment and cardiac rehabilitation programs, specific advice in clinical guidelines, and policies that promote healthy lifestyles and access to medication. Strategies targeting populations with lower educational attainment are challenging to implement [52], and can benefit from a deeper insight on RF profiles and their drivers to improve their reach and effectiveness, and eventually improve the cardiovascular health of individuals at high cardiovascular risk, particularly of those in vulnerable groups.

To our knowledge, our study was the first to describe RF clustering in secondary prevention of CHD in the Southern Cone of Latin America. In our study of the burden of multiple RF, we studied the number of RF but also the RF type and the nature of the combinations by sex and educational attainment, which may facilitate planning of prevention strategies. The limitations of our study relate to the self-reported nature of the information on CHD diagnosis, and the size of the study especially with regard to participants with higher educational level. Larger studies may be able study sex and socioeconomic disparities relating to the burden of multiple RF in more depth. Potential causes of the sex differences in burden of multiple RF (RF awareness, medication adherence or cardiac rehabilitation attendance) were beyond this analysis, but should be addressed in future studies.

## 5 Conclusion

Women had a higher burden of multiple cardiovascular RF. Cardiometabolic RF were more common than lifestyle RF, and showed a higher burden in women. Women with low educational attainment had the highest burden of multiple cardiovascular RF. Future studies should research the drivers of the higher burden cardiometabolic RF in women. Strategies and interventions for secondary prevention of CHD addressing multiple RF should be specific to sex and socioeconomic circumstances.

## Author credits

AMF, LG, PG, IV, KKG and VI contributed to the conceptualization of this manuscript. AMF conducted the formal analysis and prepared the original draft. LG cured and prepared the data. VI contributed to the acquisition of funds for this study. All co-authors reviewed and edited the manuscript, and approved the final version.

## Funding

This work was supported by the 10.13039/100000050National Heart, Lung, and Blood Institute (NHLBI) grant number HHSN268200900029C.

## Declaration of competing interest

The authors declare no conflicts of interest.
