# Supplementary material for: Risk factor clustering in men and women with CHD in the Southern Cone of Latin America
Source: Int J Cardiol Cardiovasc Risk Prev. 2023 Jan 13;16:200172. doi: 10.1016/j.ijcrp.2023.200172 (PMC9975232; doi:10.1016/j.ijcrp.2023.200172)
Supplement: Multimedia component 1 [file mmc1.docx]

**Supplementary Table 1:** Number of overall, cardiometabolic, and lifestyle RF in men and women by previous history of CHD and educational level.

| **Number of RF** | | | | | | | | | | |
| --- | --- | --- | --- | --- | --- | --- | --- | --- | --- | --- |
|  | **Participants with CHD** | | **Participants without CHD** | | **CHD and Primary school** | | **CHD and Secondary school** | | **CHD and University** | |
|  | **Female (N=317)** | **Male (N=317)** | **Female (N=4041)** | **Male N=(2846)** | **Female (N=179)** | **Male (N=164)** | **Female (N=105)** | **Male (N=108)** | **Female (N=33)** | **Male (N=45)** |
| **1** | 0 | 0,8 | 1,32 | 0,6 | 0 | 1,0 | 0 | 0,8 | 5,9 | 8,8 |
| **2** | 2,0 | 8,8 | 9,62 | 7,9 | 1,4 | 7,8 | 1,7 | 9,9 | 10,8 | 4,7 |
| **3** | 13,1 | 8,9 | 21,19 | 22,2 | 7,1 | 12,8 | 21,7 | 6,3 | 18,3 | 36,5 |
| **4** | 24,1 | 24,4 | 27,12 | 29,8 | 21,6 | 18,7 | 29,7 | 26,0 | 31,4 | 20,5 |
| **5** | 30,6 | 29,3 | 24,4 | 23,4 | 36,7 | 27,5 | 21,2 | 34,9 | 30,0 | 16,0 |
| **6** | 20,5 | 19,6 | 12,52 | 12,6 | 21,7 | 23,2 | 16,1 | 16,9 | 3,6 | 10,6 |
| **7** | 8,8 | 7,1 | 3,7 | 2,9 | 10,7 | 7,6 | 8,0 | 5,2 | 0 | 2,9 |
| **8** | 0,8 | 1,1 | 0,45 | 0,5 | 0,5 | 1,4 | 1,5 | 0 | - | - |
| **9** | - | - | 0,04 | 0,0 | - | - | - | - | - | - |
| **Number of cardiometabolic RF** | | | | | | | | | | |
| **0** | 5,1 | 11,8 | 16,8 | 14,4 | 3,2 | 12,2 | 7,1 | 10,2 | 6,5 | 14,7 |
| **1** | 18,2 | 16,7 | 28,7 | 34,1 | 12,8 | 17,2 | 26,1 | 16,4 | 15,2 | 15,9 |
| **2** | 26,3 | 33,2 | 31,7 | 30,6 | 24 | 28,6 | 29,9 | 40,3 | 24,6 | 28,5 |
| **3** | 31,5 | 24,6 | 18 | 17,1 | 38,2 | 26,2 | 23,4 | 22,5 | 29,4 | 25,1 |
| **4** | 18,9 | 13,7 | 4,7 | 3,8 | 21,8 | 15,7 | 13,6 | 10,6 | 24,2 | 15,8 |
| **Number of lifestyle RF** | | | | | | | | | | |
| **0** | 8,8 | 5,3 | 8,3 | 4,7 | 11,3 | 4,5 | 5 | 6,6 | 10,5 | 4,6 |
| **1** | 46,1 | 47 | 42 | 44,4 | 43,5 | 44,5 | 49,7 | 51,7 | 45,3 | 41,9 |
| **2** | 35,8 | 37,1 | 39,2 | 39,6 | 35,8 | 42,9 | 34,5 | 31 | 40,6 | 36,2 |
| **3** | 9,3 | 10,6 | 10 | 10,6 | 9,5 | 8,2 | 10,8 | 10,7 | 3,6 | 17,3 |
| **4** | - | - | 0,5 | 0,7 | - | - | - | - | - | - |

Results are expressed in percentages. Cardiometabolic RF include hypertension, diabetes, dyslipidemia and obesity. Lifestyle RF include current smoking, excessive alcohol consumption, low physical activity, and unhealthy diet.

**Supplementary Table 2:** Most common combinations of 3 and 4 RF in men and women by previous history of CHD and educational level.

| **Participants with CHD (N=634)** | | | | **CHD and Primary education (N=343)** | | | | **CHD and Secondary education (N=213)** | | | | **CHD and University education (N= 78)** | | | |
| --- | --- | --- | --- | --- | --- | --- | --- | --- | --- | --- | --- | --- | --- | --- | --- |
| **Comb.** | **Male**  **(N=317)** | **Female**  **(N=317)** | **p** | **Comb.** | **Male**  **(N=164)** | **Female**  **(N=179)** | **p** | **Comb.** | **Male**  **(N=108)** | **Female**  **(N=105)** | **p** | **Comb.** | **Male (N=45)** | **Female (N=35)** | **p** |
| HLOF | 28.3 | 29.6 | 0.53 | HLOF | 31.4 | 40.0 | 0.17 | HLOF | 22.8 | 21.0 | 0.76 | HLOF | 33.2 | 25.4 | 0.55 |
| HDOF | 12.5 | 14.1 | 0.30 | HDOF | 14.7 | 18.5 | 0.42 | HDOF | 10.2 | 12.3 | 0.65 | HLOP | 22.6 | 13.5 | 0.34 |
| HLOP | 11.3 | 13.8 | 0.08 | HLOP | 12.9 | 18.3 | 0.21 | HLOP | 5.1 | 14.8 | 0.02 | HDOF | 11.9 | 15.7 | 0.72 |
| LDP | 7.1 | 9.8 | 0.04 | LDP | 8.3 | 12.9 | 0.25 | LDP | 5.4 | 11.7 | 0.10 | LDP | 8.7 | 14.8 | 0.45 |
| HDOP | 5.0 | 8.1 | 0.01 | HDOP | 5.5 | 12.3 | 0.04 | HLFS | 10.7 | 4.6 | 0.12 | HDOP | 8.5 | 9.1 | 0.92 |
| HLFS | 10.6 | 7.5 | 0.00 | HLFS | 11.2 | 5.1 | 0.04 | HDOP | 3.2 | 10.5 | 0.03 | HLOS | 6.6 | 5.2 | 0.78 |
| LOFS | 6.5 | 6.1 | 0.70 | LOFS | 8.8 | 7.1 | 0.61 | AFL | 7.8 | 2.6 | 0.13 | HDOA | 7.8 | 2.3 | 0.23 |
| HDOA | 8.5 | 5.8 | 0.00 | HOFS | 8.3 | 5.2 | 0.29 | HDOA | 6.3 | 3.4 | 0.31 | AFH | 9.1 | 0.0 | 0.09 |
| HLOS | 5.7 | 5.3 | 0.70 | HDOA | 10.7 | 2.7 | 0.00 | LOFS | 4.3 | 5.5 | 0.72 | HLFS | 8.6 | 0.0 | 0.12 |
| HOFS | 5.9 | 5.0 | 0.33 | HLOS | 7.7 | 5.1 | 0.37 | HLOS | 3.1 | 4.7 | 0.51 | AFL | 5.5 | 0.0 | 0.20 |
| AFL | 4.6 | 3.1 | 0.05 | HOAS | 4.8 | 3.3 | 0.52 | HOFS | 3.4 | 4.0 | 0.81 | HOFS | 5.5 | 0.0 | 0.20 |
| AFH | 5.0 | 2.9 | 0.00 | AFH | 5.4 | 1.0 | 0.02 | HOAS | 1.6 | 2.6 | 0.59 | LOFS | 5.5 | 0.0 | 0.20 |
| HOAS | 2.8 | 2.7 | 0.93 | DOFS | 3.4 | 1.9 | 0.43 | LOPS | 0.7 | 3.2 | 0.16 | HLOA | 4.7 | 0.0 | 0.25 |
| LOPS | 1.2 | 2.2 | 0.10 | HDOS | 3.7 | 1.6 | 0.23 | HOPS | 1.5 | 2.3 | 0.65 | HDOS | 2.8 | 0.0 | 0.40 |
| HDOS | 2.1 | 1.9 | 0.72 | LOPS | 1.1 | 3.8 | 0.09 | AFH | 2.9 | 0.7 | 0.18 | HDPS | 2.8 | 0.0 | 0.40 |
| DOFS | 1.9 | 1.9 | 0.89 | HDFS | 3.5 | 1.2 | 0.18 | HDFS | 1.5 | 0.7 | 0.55 | HDFS | 2.8 | 0.0 | 0.40 |
| HOPS | 1.5 | 1.8 | 0.56 | HOPS | 1.1 | 2.5 | 0.30 | HDOS | 0.0 | 2.2 | 0.08 | HOPS | 2.8 | 0.0 | 0.40 |
| HDFS | 2.6 | 1.7 | 0.09 | AFL | 1.4 | 1.0 | 0.75 | DOFS | 0.0 | 2.1 | 0.16 | DOPS | 2.8 | 0.0 | 0.40 |
| HLOA | 1.5 | 1.2 | 0.35 | HLOA | 1.4 | 1.0 | 0.75 | HDPS | 0.7 | 0.7 | 0.97 | DOFS | 2.8 | 0.0 | 0.40 |
| DOPS | 0.4 | 0.7 | 0.56 | HLAS | 1.4 | 0.6 | 0.50 | HLOA | 0.4 | 0.7 | 0.74 | LOPS | 2.8 | 0.0 | 0.40 |
| HDPS | 0.8 | 0.6 | 0.72 | LOAS | 1.4 | 0.6 | 0.50 | HLAS | 0.9 | 0.0 | 0.19 |  |  |  |  |
| HLAS | 1.0 | 0.6 | 0.29 | HDAS | 1.4 | 0.0 | 0.14 | DOPS | 0.0 | 0.7 | 0.30 |  |  |  |  |
| LOAS | 0.8 | 0.6 | 0.42 | DOAS | 1.4 | 0.0 | 0.14 | LOAS | 0.4 | 0.0 | 0.35 |  |  |  |  |
| HDAS | 0.6 | 0.3 | 0.17 | DOPS | 0.0 | 1.2 | 0.19 |  |  |  |  |  |  |  |  |
| DOAS | 0.6 | 0.3 | 0.17 | HDPS | 0.0 | 0.5 | 0.35 |  |  |  |  |  |  |  |  |
|  |  |  |  |  |  |  |  |  |  |  |  |  |  |  |  |

Results are expressed in percentages. Abbreviations: excessive alcohol consumption (A), coronary heart disease (CHD), diabetes (D), low fruit and vegetable consumption (F), hypertension (H), dyslipidemia (L), obesity (O), low physical activity (P), smoking (S). Education: highest educational level attained by participants. Hypertension: systolic blood pressure ≥140 mm Hg, diastolic blood pressure ≥90 mm Hg, or current use of antihypertensive medication Dyslipidemia: total cholesterol ≥240 mg/dL, LDL-cholesterol ≥160 mg/Dl, HDL-cholesterol <40 mg/dL, triglyceride ≥200 mg/dL or current use of lipid-lowering medication. Diabetes: self-reported diabetes, fasting glucose ≥126 mg/dL, or current use of hypoglicemic medication. Obesity: body mass index ≥30kg/m2. Central obesity: waist circumference ≥102 cm (women); ≥88 cm (men). Excessive alcohol consumption: >14 units/week or >5 units at one occasion (around two hours more than once per month) (men); >7 units/week or >4 units in one occasion (women). Alcohol units: drink-equivalents containing 14 grams of alcohol. Low physical activity: <600 MET-minutes/week of total physical activity. Low fruit and vegetable consumption: <5 servings/day (400g/day).
